# 4-[(2,5-Dimethyl­anilino)acet­yl]-3,4-di­hydro­quinoxalin-2(1*H*)-one

**DOI:** 10.1107/S1600536809045991

**Published:** 2009-11-07

**Authors:** Waqar Nasir, Munawar Ali Munawar, Saeed Ahmad, Sohail Nadeem, Muhammad Shahid

**Affiliations:** a Institute of Chemistry, University of the Punjab, New Campus, Lahore, Pakistan; b Institute of Chemistry, Gomal University, D. I. Khan, Pakistan

## Abstract

In the title compound, C_18_H_19_N_3_O_2_, the dihedral angle between the benzene rings is 20.47 (10)° and an intra­molecular N—H⋯O hydrogen bond occurs, generating an *S*(5) ring. In the crystal, inversion dimers linked by pairs of N—H⋯O hydrogen bonds lead to *R*_2_^2^(8) loops.

## Related literature

For background to the biological activity of quinoxalines, see: Khan (2008 ▶); Miyashiro *et al.* (2009 ▶). For graph-set notation, see: Bernstein *et al.* (1995 ▶).
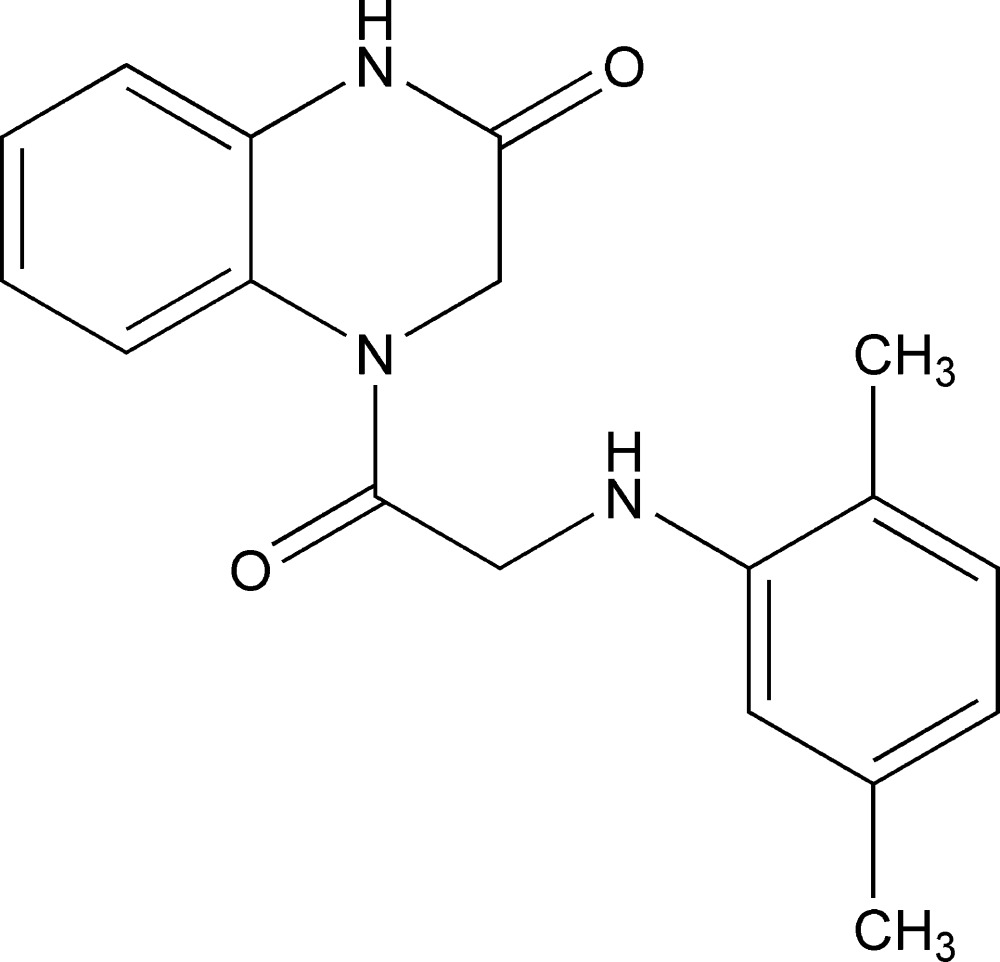


## Experimental

### 

#### Crystal data

C_18_H_19_N_3_O_2_
*M* *_r_* = 309.36
Triclinic, 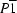

*a* = 5.3806 (2) Å
*b* = 12.3580 (6) Å
*c* = 13.2812 (6) Å
α = 62.878 (2)°
β = 84.135 (2)°
γ = 80.835 (3)°
*V* = 775.53 (6) Å^3^
*Z* = 2
Mo *K*α radiation
μ = 0.09 mm^−1^
*T* = 296 K
0.38 × 0.17 × 0.06 mm

#### Data collection

Bruker Kappa APEXII CCD diffractometer
Absorption correction: multi-scan (*SADABS*; Bruker, 2007 ▶) *T*_min_ = 0.967, *T*_max_ = 0.995
16710 measured reflections
3803 independent reflections
2277 reflections with *I* > 2σ(*I*)
*R*_int_ = 0.032

#### Refinement

*R*[*F* ^2^ > 2σ(*F* ^2^)] = 0.048
*wR*(*F* ^2^) = 0.140
*S* = 1.02
3803 reflections
216 parameters
H atoms treated by a mixture of independent and constrained refinement
Δρ_max_ = 0.21 e Å^−3^
Δρ_min_ = −0.17 e Å^−3^

### 

Data collection: *APEX2* (Bruker, 2007 ▶); cell refinement: *SAINT* (Bruker, 2007 ▶); data reduction: *SAINT*; program(s) used to solve structure: *SHELXS97* (Sheldrick, 2008 ▶); program(s) used to refine structure: *SHELXL97* (Sheldrick, 2008 ▶); molecular graphics: *ORTEP-3* (Farrugia, 1997 ▶) and *PLATON* (Spek, 2009 ▶); software used to prepare material for publication: *WinGX* (Farrugia, 1999 ▶) and *PLATON*.

## Supplementary Material

Crystal structure: contains datablocks I, global. DOI: 10.1107/S1600536809045991/hb5210sup1.cif

Structure factors: contains datablocks I. DOI: 10.1107/S1600536809045991/hb5210Isup2.hkl

Additional supplementary materials:  crystallographic information; 3D view; checkCIF report

## Figures and Tables

**Table 1:** Hydrogen-bond geometry (Å, °)

*D*—H⋯*A*	*D*—H	H⋯*A*	*D*⋯*A*	*D*—H⋯*A*
N3—H2*N*⋯O2	0.85 (2)	2.20 (2)	2.620 (2)	110.0 (16)
N1—H1*N*⋯O1^i^	0.91 (2)	1.93 (2)	2.8405 (19)	176.9 (18)

Symmetry code: (i) 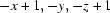.

**Fig. 1.**
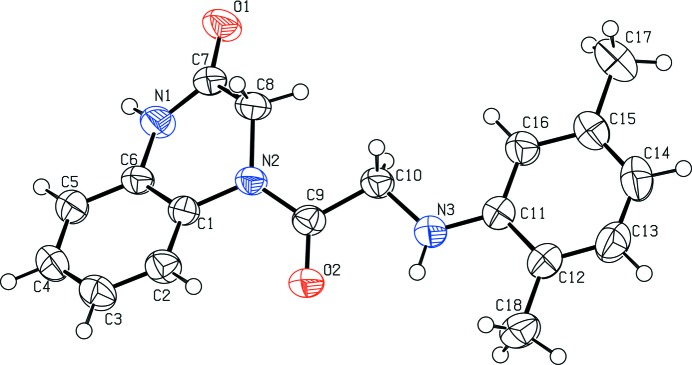
The molecular structure of (I) showing 50% displacement ellipsoids.

**Fig. 2.**
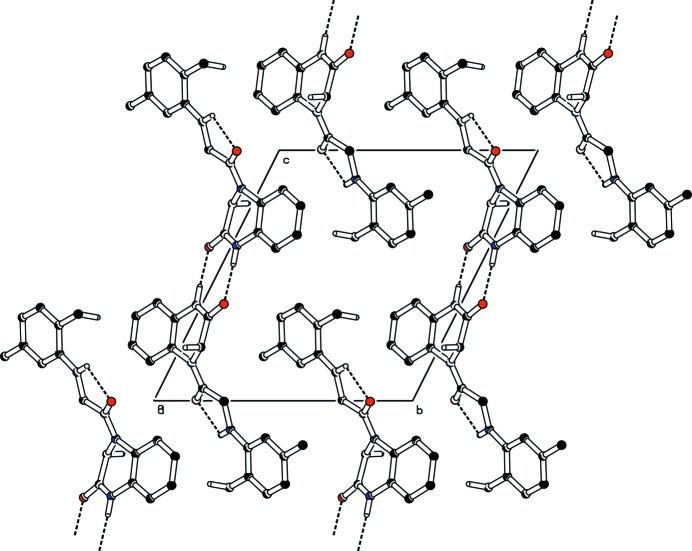
Packing diagram for (I) showing the intermolecular and intramolecular hydrogen bonding using dashed lines. The hydrogen atoms not involved in hydrogen bonding have been omitted for clarity.

## supplementary crystallographic information

### Comment

Quinoxalines represent an important class of nitrogen hetrocycles possessing wide range of biological activities (e.g. Khan, 2008). Moreover, several quinoxalines have been reported as inhibitors of, e.g. poly(ADP-ribose)polymerase-1 (Miyashiro *et al.*, 2009). During our research we tried to synthesize novel quinoxaline derivatives which may possess enhanced biological activities.

The pyrazinone ring in the quinoxaline system have gained envelop shape to some extent with the r.m.s. deviation (0.1637 A°). The intramolecular hydrogen bonding present in the molecule give rise to the formation of five membered ring motif S(5) (Bernstein, *et al.*, 1995) which is oriented at a dihedral angle of 28.13 (0.24) ° with respect to pyrazinone ring. The dihedral angle between planar *p*-xylene and pyrazinone ring is 21.31 (0.09) °. Further more symmetry related N–H···O type hydrogen bonding helps to stabilize the crystal structure of the molecule by forming eight membered ring motif *R*_2_^2^8.

### Experimental

2,5 Dimethyl aniline (0.67 g, 5.57 mmol) added to the suspension of 4-(Chloroacetyl)-3,4-dihydroquinoxalin-2 (1*H*)-one (1.25 g, 5.57 mmol and NaHCO_3_ (0.7 g, 8.35 mmol) in 2-propanol. The reaction mixture was refluxed for 8 h. Then reaction was monitored by TLC (CHCl_3_ and ethylacetate). The solid obtained on cooling was filtered, washed with chloroform and methanol. Colourless chunks of (I) were grown from an acetone DMF mixture by slow evaporation at room temperature.

### Refinement

The H-atoms for the aromatic (C—H = 0.93), methylene (C—H = 0.97) and methyl carbon (C—H = 0.96) atoms were geometrically placed and treated as riding atoms: with *U*_iso_(H) = 1.2*U*_eq_ for aromatic and methylene carbon atoms and *U*_iso_(H) = 1.5*U*_eq_ for methyl carbon atoms. The (N—H 0.85 (2)–0.91 (2) atoms were freely refined with *U*_iso_(H) = 1.2*U*_eq_ (parent N-atom)

### Crystal data

**Table:**

C_18_H_19_N_3_O_2_	*Z* = 2
*M**_r_* = 309.36	*F*(000) = 328
Triclinic, *P*1	*D*_x_ = 1.325 Mg m^−^^3^
Hall symbol: -P 1	Mo *K*α radiation, λ = 0.71073 Å
*a* = 5.3806 (2) Å	Cell parameters from 3605 reflections
*b* = 12.3580 (6) Å	θ = 3.1–25.1°
*c* = 13.2812 (6) Å	µ = 0.09 mm^−^^1^
α = 62.878 (2)°	*T* = 296 K
β = 84.135 (2)°	Chunk, colourless
γ = 80.835 (3)°	0.38 × 0.17 × 0.06 mm
*V* = 775.53 (6) Å^3^	

### Data collection

**Table:**

Bruker Kappa APEXII CCD diffractometer	3803 independent reflections
Radiation source: fine-focus sealed tube	2277 reflections with *I* > 2σ(*I*)
graphite	*R*_int_ = 0.032
φ and ω scans	θ_max_ = 28.3°, θ_min_ = 3.1°
Absorption correction: multi-scan (*SADABS*; Bruker, 2007)	*h* = −7→7
*T*_min_ = 0.967, *T*_max_ = 0.995	*k* = −16→16
16710 measured reflections	*l* = −17→17

### Refinement

**Table:**

Refinement on *F*^2^	Primary atom site location: structure-invariant direct methods
Least-squares matrix: full	Secondary atom site location: difference Fourier map
*R*[*F*^2^ > 2σ(*F*^2^)] = 0.048	Hydrogen site location: inferred from neighbouring sites
*wR*(*F*^2^) = 0.140	H atoms treated by a mixture of independent and constrained refinement
*S* = 1.02	*w* = 1/[σ^2^(*F*_o_^2^) + (0.0583*P*)^2^ + 0.1485*P*] where *P* = (*F*_o_^2^ + 2*F*_c_^2^)/3
3803 reflections	(Δ/σ)_max_ < 0.001
216 parameters	Δρ_max_ = 0.21 e Å^−^^3^
0 restraints	Δρ_min_ = −0.17 e Å^−^^3^

### Special details

**Table:**

Geometry. All s.u.'s (except the s.u. in the dihedral angle between two l.s. planes) are estimated using the full covariance matrix. The cell s.u.'s are taken into account individually in the estimation of s.u.'s in distances, angles and torsion angles; correlations between s.u.'s in cell parameters are only used when they are defined by crystal symmetry. An approximate (isotropic) treatment of cell s.u.'s is used for estimating s.u.'s involving l.s. planes.
Refinement. Refinement of *F*^2^ against ALL reflections. The weighted *R*-factor *wR* and goodness of fit *S* are based on *F*^2^, conventional *R*-factors *R* are based on *F*, with *F* set to zero for negative *F*^2^. The threshold expression of *F*^2^ > 2σ(*F*^2^) is used only for calculating *R*-factors(gt) *etc*. and is not relevant to the choice of reflections for refinement. *R*-factors based on *F*^2^ are statistically about twice as large as those based on *F*, and *R*- factors based on ALL data will be even larger.

### Fractional atomic coordinates and isotropic or equivalent isotropic displacement parameters (Å^2^)

**Table:**

	*x*	*y*	*z*	*U*_iso_*/*U*_eq_	
C1	0.3267 (3)	−0.03248 (15)	0.21118 (13)	0.0375 (4)	
C2	0.2304 (3)	−0.09075 (17)	0.15875 (15)	0.0474 (4)	
H2	0.2528	−0.0632	0.0809	0.057*	
C3	0.1012 (4)	−0.18983 (18)	0.22234 (17)	0.0565 (5)	
H3	0.0350	−0.2283	0.1870	0.068*	
C4	0.0695 (4)	−0.23204 (18)	0.33759 (17)	0.0595 (5)	
H4	−0.0191	−0.2984	0.3798	0.071*	
C5	0.1690 (3)	−0.17607 (17)	0.39049 (15)	0.0527 (5)	
H5	0.1486	−0.2050	0.4685	0.063*	
C6	0.2993 (3)	−0.07679 (15)	0.32755 (13)	0.0402 (4)	
C7	0.5923 (3)	0.04788 (16)	0.33477 (14)	0.0432 (4)	
C8	0.6625 (3)	0.07691 (18)	0.21398 (13)	0.0478 (5)	
H8A	0.7148	0.1581	0.1760	0.057*	
H8B	0.8051	0.0190	0.2119	0.057*	
C9	0.3822 (3)	0.16519 (16)	0.05055 (13)	0.0394 (4)	
C10	0.5334 (3)	0.27254 (16)	−0.00272 (13)	0.0441 (4)	
H10A	0.5118	0.3170	0.0424	0.053*	
H10B	0.7110	0.2433	−0.0060	0.053*	
C11	0.5571 (3)	0.45478 (16)	−0.18728 (14)	0.0446 (4)	
C12	0.4976 (3)	0.51241 (17)	−0.30190 (15)	0.0487 (4)	
C13	0.6002 (4)	0.61824 (19)	−0.37152 (17)	0.0605 (5)	
H13	0.5629	0.6575	−0.4478	0.073*	
C14	0.7563 (4)	0.66854 (19)	−0.33251 (18)	0.0630 (6)	
H14	0.8178	0.7415	−0.3819	0.076*	
C15	0.8211 (3)	0.61092 (17)	−0.22080 (17)	0.0521 (5)	
C16	0.7209 (3)	0.50364 (17)	−0.14910 (15)	0.0487 (4)	
H16	0.7643	0.4633	−0.0735	0.058*	
C17	0.9978 (4)	0.6614 (2)	−0.1771 (2)	0.0713 (6)	
H17A	0.9490	0.6454	−0.1005	0.107*	
H17B	0.9907	0.7483	−0.2235	0.107*	
H17C	1.1664	0.6229	−0.1795	0.107*	
C18	0.3267 (4)	0.4598 (2)	−0.34590 (16)	0.0609 (5)	
H18A	0.3982	0.3782	−0.3324	0.091*	
H18B	0.3080	0.5101	−0.4257	0.091*	
H18C	0.1648	0.4573	−0.3077	0.091*	
N1	0.4033 (3)	−0.02041 (14)	0.38128 (12)	0.0479 (4)	
N2	0.4571 (2)	0.07258 (12)	0.15222 (11)	0.0388 (3)	
N3	0.4475 (3)	0.35148 (15)	−0.11433 (13)	0.0580 (5)	
O1	0.7078 (2)	0.08343 (13)	0.38634 (10)	0.0589 (4)	
O2	0.2018 (2)	0.16278 (12)	0.00335 (10)	0.0550 (4)	
H1N	0.366 (4)	−0.0432 (18)	0.4561 (19)	0.066*	
H2N	0.339 (4)	0.3255 (19)	−0.1377 (17)	0.066*	

### Atomic displacement parameters (Å^2^)

**Table:**

	*U*^11^	*U*^22^	*U*^33^	*U*^12^	*U*^13^	*U*^23^
C1	0.0355 (8)	0.0409 (9)	0.0372 (9)	−0.0074 (7)	−0.0020 (6)	−0.0174 (7)
C2	0.0535 (10)	0.0532 (11)	0.0437 (10)	−0.0114 (8)	−0.0022 (8)	−0.0272 (9)
C3	0.0680 (12)	0.0528 (11)	0.0600 (12)	−0.0194 (10)	−0.0072 (9)	−0.0305 (10)
C4	0.0696 (13)	0.0527 (12)	0.0557 (12)	−0.0274 (10)	−0.0070 (10)	−0.0167 (10)
C5	0.0595 (11)	0.0560 (12)	0.0393 (10)	−0.0209 (9)	−0.0047 (8)	−0.0137 (9)
C6	0.0400 (9)	0.0454 (10)	0.0383 (9)	−0.0101 (7)	−0.0055 (7)	−0.0191 (8)
C7	0.0443 (9)	0.0476 (10)	0.0390 (9)	−0.0101 (8)	−0.0091 (7)	−0.0175 (8)
C8	0.0396 (9)	0.0648 (12)	0.0395 (10)	−0.0183 (8)	−0.0044 (7)	−0.0194 (9)
C9	0.0400 (9)	0.0481 (10)	0.0342 (9)	−0.0092 (7)	−0.0001 (7)	−0.0211 (8)
C10	0.0457 (9)	0.0514 (11)	0.0371 (9)	−0.0142 (8)	−0.0029 (7)	−0.0187 (8)
C11	0.0459 (10)	0.0438 (10)	0.0424 (10)	−0.0066 (8)	−0.0030 (7)	−0.0173 (8)
C12	0.0508 (10)	0.0496 (11)	0.0418 (10)	−0.0020 (8)	−0.0040 (8)	−0.0181 (8)
C13	0.0656 (13)	0.0581 (12)	0.0431 (11)	−0.0049 (10)	−0.0028 (9)	−0.0106 (9)
C14	0.0669 (13)	0.0493 (12)	0.0608 (13)	−0.0165 (10)	0.0107 (10)	−0.0142 (10)
C15	0.0487 (10)	0.0501 (11)	0.0614 (12)	−0.0123 (8)	0.0078 (9)	−0.0284 (10)
C16	0.0520 (10)	0.0515 (11)	0.0437 (10)	−0.0136 (9)	−0.0019 (8)	−0.0200 (9)
C17	0.0663 (13)	0.0734 (15)	0.0898 (17)	−0.0316 (11)	0.0121 (12)	−0.0459 (13)
C18	0.0656 (12)	0.0696 (14)	0.0475 (11)	−0.0066 (10)	−0.0132 (9)	−0.0247 (10)
N1	0.0548 (9)	0.0629 (10)	0.0327 (8)	−0.0240 (8)	0.0006 (6)	−0.0224 (7)
N2	0.0381 (7)	0.0471 (8)	0.0331 (7)	−0.0131 (6)	−0.0024 (5)	−0.0169 (6)
N3	0.0679 (11)	0.0545 (10)	0.0456 (9)	−0.0250 (8)	−0.0165 (8)	−0.0090 (8)
O1	0.0667 (8)	0.0757 (9)	0.0453 (7)	−0.0334 (7)	−0.0050 (6)	−0.0283 (7)
O2	0.0546 (7)	0.0619 (8)	0.0449 (7)	−0.0206 (6)	−0.0148 (6)	−0.0140 (6)

### Geometric parameters (Å, °)

**Table:**

C1—C6	1.385 (2)	C10—H10A	0.9700
C1—C2	1.386 (2)	C10—H10B	0.9700
C1—N2	1.4290 (19)	C11—N3	1.383 (2)
C2—C3	1.380 (2)	C11—C16	1.392 (2)
C2—H2	0.9300	C11—C12	1.402 (2)
C3—C4	1.375 (3)	C12—C13	1.376 (3)
C3—H3	0.9300	C12—C18	1.502 (2)
C4—C5	1.377 (2)	C13—C14	1.382 (3)
C4—H4	0.9300	C13—H13	0.9300
C5—C6	1.384 (2)	C14—C15	1.376 (3)
C5—H5	0.9300	C14—H14	0.9300
C6—N1	1.402 (2)	C15—C16	1.388 (2)
C7—O1	1.2233 (18)	C15—C17	1.504 (3)
C7—N1	1.341 (2)	C16—H16	0.9300
C7—C8	1.496 (2)	C17—H17A	0.9600
C8—N2	1.4632 (18)	C17—H17B	0.9600
C8—H8A	0.9700	C17—H17C	0.9600
C8—H8B	0.9700	C18—H18A	0.9600
C9—O2	1.2192 (17)	C18—H18B	0.9600
C9—N2	1.361 (2)	C18—H18C	0.9600
C9—C10	1.514 (2)	N1—H1N	0.91 (2)
C10—N3	1.429 (2)	N3—H2N	0.85 (2)
			
C6—C1—C2	119.61 (15)	C16—C11—C12	119.56 (16)
C6—C1—N2	116.51 (13)	C13—C12—C11	117.64 (17)
C2—C1—N2	123.89 (15)	C13—C12—C18	121.72 (17)
C3—C2—C1	119.81 (16)	C11—C12—C18	120.64 (16)
C3—C2—H2	120.1	C12—C13—C14	122.58 (18)
C1—C2—H2	120.1	C12—C13—H13	118.7
C4—C3—C2	120.47 (16)	C14—C13—H13	118.7
C4—C3—H3	119.8	C15—C14—C13	120.20 (18)
C2—C3—H3	119.8	C15—C14—H14	119.9
C3—C4—C5	120.01 (17)	C13—C14—H14	119.9
C3—C4—H4	120.0	C14—C15—C16	118.20 (17)
C5—C4—H4	120.0	C14—C15—C17	121.24 (18)
C4—C5—C6	119.99 (17)	C16—C15—C17	120.56 (18)
C4—C5—H5	120.0	C15—C16—C11	121.77 (17)
C6—C5—H5	120.0	C15—C16—H16	119.1
C5—C6—C1	120.08 (15)	C11—C16—H16	119.1
C5—C6—N1	120.15 (15)	C15—C17—H17A	109.5
C1—C6—N1	119.77 (14)	C15—C17—H17B	109.5
O1—C7—N1	123.53 (16)	H17A—C17—H17B	109.5
O1—C7—C8	120.51 (15)	C15—C17—H17C	109.5
N1—C7—C8	115.94 (14)	H17A—C17—H17C	109.5
N2—C8—C7	113.34 (13)	H17B—C17—H17C	109.5
N2—C8—H8A	108.9	C12—C18—H18A	109.5
C7—C8—H8A	108.9	C12—C18—H18B	109.5
N2—C8—H8B	108.9	H18A—C18—H18B	109.5
C7—C8—H8B	108.9	C12—C18—H18C	109.5
H8A—C8—H8B	107.7	H18A—C18—H18C	109.5
O2—C9—N2	122.03 (15)	H18B—C18—H18C	109.5
O2—C9—C10	120.83 (15)	C7—N1—C6	122.90 (15)
N2—C9—C10	117.14 (13)	C7—N1—H1N	116.9 (12)
N3—C10—C9	108.72 (12)	C6—N1—H1N	118.9 (12)
N3—C10—H10A	109.9	C9—N2—C1	122.08 (12)
C9—C10—H10A	109.9	C9—N2—C8	123.03 (13)
N3—C10—H10B	109.9	C1—N2—C8	114.71 (13)
C9—C10—H10B	109.9	C11—N3—C10	122.78 (14)
H10A—C10—H10B	108.3	C11—N3—H2N	120.7 (14)
N3—C11—C16	121.54 (16)	C10—N3—H2N	115.9 (14)
N3—C11—C12	118.89 (16)		
			
C6—C1—C2—C3	2.1 (3)	C13—C14—C15—C17	177.88 (19)
N2—C1—C2—C3	−178.28 (16)	C14—C15—C16—C11	−0.6 (3)
C1—C2—C3—C4	−0.7 (3)	C17—C15—C16—C11	−179.95 (17)
C2—C3—C4—C5	−0.6 (3)	N3—C11—C16—C15	−176.58 (18)
C3—C4—C5—C6	0.5 (3)	C12—C11—C16—C15	2.3 (3)
C4—C5—C6—C1	0.9 (3)	O1—C7—N1—C6	−169.54 (17)
C4—C5—C6—N1	−179.29 (17)	C8—C7—N1—C6	8.9 (3)
C2—C1—C6—C5	−2.2 (3)	C5—C6—N1—C7	158.51 (17)
N2—C1—C6—C5	178.14 (15)	C1—C6—N1—C7	−21.7 (3)
C2—C1—C6—N1	178.03 (16)	O2—C9—N2—C1	−1.1 (3)
N2—C1—C6—N1	−1.6 (2)	C10—C9—N2—C1	179.09 (14)
O1—C7—C8—N2	−157.17 (17)	O2—C9—N2—C8	−175.90 (16)
N1—C7—C8—N2	24.4 (2)	C10—C9—N2—C8	4.3 (2)
O2—C9—C10—N3	−7.3 (2)	C6—C1—N2—C9	−140.30 (16)
N2—C9—C10—N3	172.49 (15)	C2—C1—N2—C9	40.0 (2)
N3—C11—C12—C13	177.03 (18)	C6—C1—N2—C8	34.9 (2)
C16—C11—C12—C13	−1.9 (3)	C2—C1—N2—C8	−144.75 (17)
N3—C11—C12—C18	−2.7 (3)	C7—C8—N2—C9	129.03 (17)
C16—C11—C12—C18	178.41 (17)	C7—C8—N2—C1	−46.1 (2)
C11—C12—C13—C14	−0.2 (3)	C16—C11—N3—C10	−14.3 (3)
C18—C12—C13—C14	179.55 (19)	C12—C11—N3—C10	166.82 (18)
C12—C13—C14—C15	1.9 (3)	C9—C10—N3—C11	−176.65 (17)
C13—C14—C15—C16	−1.5 (3)		

### Hydrogen-bond geometry (Å, °)

**Table:**

*D*—H···*A*	*D*—H	H···*A*	*D*···*A*	*D*—H···*A*
N3—H2N···O2	0.85 (2)	2.20 (2)	2.620 (2)	110.0 (16)
N1—H1N···O1^i^	0.91 (2)	1.93 (2)	2.8405 (19)	176.9 (18)

Symmetry codes: (i) −*x*+1, −*y*, −*z*+1.
